# Medical need and health need

**DOI:** 10.1177/14777509231173561

**Published:** 2023-05-08

**Authors:** Ben Davies

**Affiliations:** Uehiro Centre for Practical Ethics, 6396 University of Oxford, Oxford, UK

**Keywords:** Medical need, medical necessity, distributive justice

## Abstract

I introduce a distinction between health need and medical need, and raise several questions about their interaction. Health needs are needs that relate directly to our health condition. Medical needs are needs which bear some relation to medical institutions or processes. I suggest that the question of whether medical insurance or public care should cover medical needs, health needs, or only needs which fit both categories is a political question that cannot be resolved definitionally. I also argue against an overly strict definition of medical need on the grounds that this presupposes, wrongly, that medical intervention should always be a last resort.

## Introduction

Allocations of healthcare on the basis of ability to pay are often contrasted by opponents with the more progressive-sounding idea that healthcare should be allocated on the basis of ‘medical need’.^1–5^ But many discussions of medical need emphasise either the difficulty in defining it, or the fact that failure to define it has not served as an obstacle to wide use,^4,6–9^ though some have suggested abandoning the term.^10^ The use of the term is understandable: needs-claims are often taken to have a ‘moral edge’ that claims on other grounds – such as desire – do not.^11^

That needs-claims operate in real-world contexts means we should be cautious in insisting on too much conceptual precision; what matters most is whether the language of needs serves useful purposes well. But there is clearly a danger that a vague term which is understood differently by different groups and individuals leads to inconsistency and injustice. First, if medical need is not clearly defined, then it is *more vulnerable* (not inevitably open) to well-resourced and more powerful groups having their claims successfully categorised as medical needs, and less powerful and poorly resourced groups being neglected. Second, patients have a (defeasible) entitlement to be able to understand the systems and processes that have fundamental effects on their lives. A poorly defined concept that is open to manipulation is less likely to serve either of these important goals.

My aim in this article is not to offer a definitive definition of medical need, but instead to raise two distinctions that we might make which raise questions about the scope of medical need. Speaking roughly, these distinctions respectively concern the ‘medical’ aspect of medical need, and the ‘need’ aspect. Concerning the term ‘medical’, I distinguish between medical need and what I call ‘health need’. Whereas health needs are those needs we have with respect to our health, my definition of medical need concerns which sorts of social institutions are best placed to meet it. I consider whether either of these ideas is a subset of the other – that is, are all health needs medical needs, or are all medical needs health needs? I then consider some implications of this distinction for practical priority-setting, arguing that the relevant considerations are primarily political rather than conceptual: in other words, while the idea of medical need might help our decision making, it cannot avoid difficult political decisions. Finally, concerning the term ‘need’, I critically discuss an influential view of medical need which assumes that we can say something is a medical need (in a normatively significant sense) only if other approaches have been tried first; I draw on this discussion to argue that we should take a fairly liberal view of the role medical institutions, rejecting the claim that medical needs be restricted to those which can *only* be addressed by medical means, and questioning whether we should even insist that medical needs are those which can *best* be alleviated by medical means.

## Medical need and health need

L Chad Horne^12^ distinguishes between the idea that healthcare should be concerned with ‘medically necessary’ interventions, and that people should be able to meet their (basic) medical needs. The latter of these is more reflective of the above contrast between allocation by need and ability to pay. On the former view, which Horne endorses, medical need is equivalent to *clinical effectiveness*: a *treatment* is medically necessary when it would successfully treat a medical condition.

As Horne suggests, however, his understanding of allocation according to need is not all that plausible if understood in absolute terms. There are some services – most obviously services in reproductive health such as nonmedically indicated abortion or vasectomy – which are not clinically effective in the sense of preventing a health issue. Yet this fact alone does not settle the question of whether such services should be provided by the healthcare service.

One distinction that may be of use here is between *medical* need and *health* need. Unfortunately, the distinction I suggest introduces a further, disparate definition of medical need. As such, this distinction should probably not be thrown unaided into the real-world of healthcare priority-setting; different, less confusing terminology might need to be found.

On this framing, whether something is a medical need concerns the involvement of medical institutions or processes in addressing it.^3^ There are several options, of varying degrees of strictness. On the strictest possible interpretation, a medical need is a need that could only be addressed by medical means. A middle-ground interpretation is that medical needs are those which are *best* addressed by medical means, but which could be addressed by other means. Finally, the broadest available definition casts medical needs as those which could be addressed by medical means, even if medical means are not the *best* means.

Consider, for instance, the currently pressing question of individuals who are struggling to afford their energy bills during the winter. In the United Kingdom, primary physicians working for the publicly funded National Health Service (NHS) in some areas can ‘prescribe’ that someone receives contribution towards energy bills if they also have one of a range of lung conditions.^13^ Thus, a need is addressed by institutional medicine. However, it is not addressed by medical means, and it is not a need that could *only* be addressed by institutional medicine: we might instead have a system where anyone who was struggling with their energy bills to the point where they were unable to run their heating received considerable support in doing so without having to mediate through a medical professional. And it is thus debatable whether this is an issue which is *best* addressed by institutional medicine. Whether being unable to afford to put the heating on is a ‘medical’ need, then, depends on how strictly we define the relationship between meeting the need and medical processes, institutions, and professionals.

In contrast, a health need is a need that directly concerns an individual's health. For instance, we might conceptualise health needs as shortfall from optimal health (see Caulfield and Zarzeczny^6^ for discussion); *currently treatable* shortfall from optimal health^14^; or as shortfall from some less demanding sub-optimal level of health. Health need might also incorporate considerations such as the urgency of treatment, that is, how long a patient has left before some severe, perhaps irreversible or much more complex-to-treat health condition sets in.^15,16^ Different versions of this view will be more or less helpful in allocating healthcare resources. For instance, if health need is taken to include *any* shortfall from *optimal* health, the concept by itself will do little to direct resource allocation beyond excluding non-health needs, and will require supplementation by considerations such as severity and urgency.^3,12^

Two questions emerge as a result of this framing. At least on narrower framings of health needs, it is clear that not all health needs are medical needs: some people's health needs are best addressed by non-medical means. But what about the converse relation: Are all medical needs health needs? On a wide definition of medical need – that is, as any need which *could in principle* be tackled by medical means – the answer is no. Some conditions which are not health needs could in principle be treated by medical means, or by medical institutions. If we adopt a narrower definition of medical need, either as needs which are best treated by medical means, or as needs which are *only* amenable to medical intervention, things are less clear. Are there non-health conditions that are best treated, or only treatable, by medical means?

Several possible examples concern reproductive health, though some of these are more plausible than others. For instance, some claim that pregnancy is not a health condition,^4,17,18^ and that abortion and pregnancy care are not justifiable by reference to health need. However, both these cases could in principle be seen as forms of preventive public health; a primary reason for having healthcare professionals involved in pregnancy care, birth, and abortion are that these areas often give rise to health needs that it is best to have medical professionals monitor from the outset. Other cases involve no health condition *per se,* but concern procedures which, if not carried out in a medical context, present greater risk of ill health if they go wrong. Examples include vasectomy and circumcision. In these cases, the justification for making these *medical* procedures may still ultimately rest on the grounds of health.

Alternatively, Norman Daniels and James E. Sabin^19^ raise the example of using a medical procedure, psychotherapy, for problems which nobody would regard as a health need, such as marital unhappiness. In their example the ‘patient’ (the ‘Unhappy Husband’) finds engagement with a therapist productive, and wishes it would be covered by insurance. But he accepts that he does not have what I have called a ‘health need’, and thinks it would be unfair for his sessions to be covered. Thus, as Daniels and Sabin present it, this is potentially a case of medical need without health need. On their view, this is sufficient to mean that it should not be covered by a health insurance package (though they do not claim that the medical system should not engage with this case).

The second question raised by the distinction is whether medical systems, professionals, and procedures should be concerned with health needs, medical needs, or only with cases which are both.

For instance, Chris Kaposy^17^ argues that while a case can be made for abortion being (in my terminology) a health need, the justification for including abortion in public health funding should rely not on whether the aim of an intervention is strictly health-related, but rather on whether the *means* to that end are ‘healthcare procedures’.

Translated into the terminology of this article, Kaposy argues that public health funding should focus on medical needs rather than health needs, thus including abortion care even if it is not a matter of health. Interestingly, Kaposy's argument would seem to exclude cases like the primary care heating prescription, since having one's heating bills subsidised is not a ‘healthcare procedure’, even though what is being addressed is, at least explicitly, a health need. He thus seems to draw a moderately demanding definition of particular interventions being *actually addressed* by healthcare *procedures*, not merely the healthcare system, and not merely in principle addressable.

On this view, then, it is neither necessary nor sufficient that someone has a health need for it to be the subject of health-related funding; whereas it does seem to be at least necessary, and perhaps sufficient, that a situation is addressed by medical procedures.

One argument in favour of this is that due to the dynamic relationship between health and other goods, almost anything *could* be classified as a health need,^20^ and one might worry about overloading healthcare systems with problems that should really be the preserve of other government departments. One pragmatic reason for this process may be that health budgets are often politically harder to cut than the budgets of other departments; but while there is much to be said for joined-up thinking that recognises the health implications of housing, education and transport, there is clear risk in giving a single system too much to do.

On the other hand, if we take as given that governments are less willing to cut health budgets, there is an argument in favour of pushing to recognise various disparate interventions as ‘health’ interventions, which is simply that they are thus more likely to be taken seriously.^21^ As Daniel Skinner^4^ notes, the concept of ‘medical need’, sometimes assumed to be objectively ascertainable, thus becomes a political matter of how much we can cram in – or, if we are aiming to resist spending increases, keep out – to best suit our priorities.^22^

Contrast Kaposy's argument with Horne's discussion – mentioned above – of whether interventions are medically necessary, which is concerned *both* with what I have termed medical and health need. By virtue of focusing on medical interventions, Horne is necessarily concerned with medical need, while the justification for including a condition under medical insurance is that it treats a ‘pathological condition’,^a^ and thus is focused on health need.

Horne argues that these restrictions are justified by the purpose of health insurance: people buy health insurance to ‘reduce the uncertainty regarding their future [health] needs’, and inclusion of non-health-related considerations would increase uncertainty. Horne's primary concern is with whether healthcare allocation should focus on one's *overall* situation; for instance, should those who have additional, non-health needs due to poverty or social injustice be higher priorities for care? Horne argues that including such factors increases uncertainty, since it is much more difficult for people to predict what their insurance would cover. However, this argument primarily concerns the inclusion of non-health factors in deciding whether, and with what priority, to treat different patients. It is less clear that this problem arises with the inclusion of specific non-health needs in the pool of what the medical system will address, so long as their inclusion is publicised and explicitly justified. It is also less clear whether Horne's argument applies to public systems such as the NHS.

Thus, there are reasonable arguments for various positions around whether healthcare systems should focus on medical need, health need or both. However, the relevant considerations are primarily political rather than conceptual; once we distinguish between medical and health need, definitional appeals have little purchase. While appeals to medical (and potentially health) need may serve as useful heuristics in decision making, and while technical definitions of medical need can of course be used in determining the priority of claims, these are necessarily stipulative and, to the extent that their stipulated content is not made explicit, prone to political manipulation.

## Medical need and medical benefit

Let me return finally to the issue of how categorising something as a medical need should relate to its amenability to medical means. I considered a few options, namely that something might be classed as a medical need, variously, if it *could* be addressed by medical means; if it is *best* addressed by medical means; or if it is *only* addressable by medical means (following Kaposy we might add a fourth option, which is whether it is *in fact addressed* by medical means in the relevant jurisdiction, but I ignore this possibility since it seems insufficiently responsive to normative concerns). A final question I consider is around the order of priority between addressing a particular need (whether it falls under my definition of ‘health’ need or not) through medical or non-medical means.

To begin with, we might prefer a stricter definition of medical need (i.e., moving along the spectrum away from ‘in principle addressable’ towards ‘only addressable’). But as I will suggest, this will require a further assumption to be justified.

Consider, for instance, the following quotation from Daniels and Sabin,^19^ where they justify their proposal that healthcare be used only to restore people to ‘normal functioning’. In the terminology of this article, their proposal is that medical services should focus on needs which are *both* health and medical needs, defining health needs quite narrowly, though they allow that if there were broad social consensus for expanding the purpose of healthcare this would be acceptable. Considering whether shyness should be a legitimate subject of medical treatment, they describe their preferred model as insisting that:

*Health care is not the only agent of social responsibility. People suffering from lack of social skill can be ministered to by education, training, families, religious and community groups, and other social institutions … health care insurance coverage should be restricted to disadvantages caused by disease and disability unless society explicitly decides to use it to mitigate other forms of disadvantage as well.*

Similarly, consider Rem B. Edwards’ (critical) discussion of pain management,^23^ where some medical professionals may try to resist giving pain medication to patients who have not first tried to manage their own pain through resilience and will power.

Finally Lynette Reid^3^ endorses an account of medical need that draws on David Wiggins's broader philosophical account of need. On Wiggins's^24^ view, needs can be more or less ‘entrenched’ according to various factors including how much harm will be caused if they are left unmet (severity), how long is available before this harm will come to pass (urgency), and most crucially for my purposes whether there are alternative ways to meet the need, which Wiggins terms ‘substitutability’. According to Reid^3^ ‘healthcare needs are entrenched needs’, which I take to include but not be fully determined by how substitutable they are. As Reid puts it^3^ ‘There are many ways to sate hunger but few ways to treat a specific cancer’. The converse presumption is that if a need is *not* entrenched, then it cannot be a healthcare need. Although Reid does not say so, I will interpret this as implying that where a need can be met by non-medical means, this at least weakens the case for its being met by medical means (a similar idea to Daniels’ and Sabin's view). Similarly, the view Edwards criticises seems to be that if pain can be managed by non-medical means (individual fortitude) then it should not be managed by medical means, and is thus not a genuine *medical* need.

On one interpretation, this implies the strictest of my three options for defining medical need: that medical needs are those which can *only* be addressed through medical means. This argument will work only if we presume a further claim, which is that medical interventions should be a last resort.

To see why, consider an argument which parallels Daniels and Sabin's, but this time focused on whether individuals who are shy in ways that causes them to suffer should be assisted through educational means. Changes from the original text are in bold:

***Education and training*** *are not the only agent of social responsibility. People suffering from lack of social skill can be ministered to by **health care**, families, religious and community groups, and other social institutions…*

Thus, if we apply the same standards, we might also insist that crippling shyness is not an ‘educational need’. And we can cycle through each possible approach to show that shyness is not a ‘religious need’ or a ‘community need’ or a ‘family need’. If the same standard is applied in each context, then crippling shyness turns out not to be a need at all, since it is not *solely* addressable by any particular institutional or social intervention.

Of course, if we have a further argument that medical interventions should always be the last type of intervention considered, then the above problem does not arise. We are not then licensed to make the same argument around the availability of alternatives for educational or other types of intervention.

However, it is not plausible that medical intervention should *always* be the last resort. While there are often good reasons to be careful about advocating medical interventions, there are also risks to other types of intervention. And sometimes it is predictable – as in Edwards's case of pain management – that non-medical means will do a worse job, or be much more costly for the needy individual.

Thus, I suggest that we should not adopt the strictest possible definition of medical needs, as those which are only amenable to medical intervention. We should at most adopt the moderate view, which defines medical need in terms of what is *best* addressed by medical intervention. And in fact, even this may be too strict. For instance, we might know that a non-medical intervention would be better for a particular need than the best available medical intervention, but we also know that that non-medical intervention is not currently feasible. In that case, it would not be the case that the need is *best* addressed by medical intervention, but there may still be a strong case for addressing it through medical intervention. Whether this takes us all the way to defining medical need in the loosest sense, as needs which could in principle be addressed through medical means, is unclear; perhaps there is a better definition between these two options.

## Conclusion

I introduced a distinction between health need and medical need, and raised several questions about their interaction. Health needs are needs that relate directly to our health condition. Medical needs are needs which bear some relation to medical institutions or processes. I suggested that the question of whether medical insurance or public care should cover medical needs, health needs or only needs which fit both categories is a political question that cannot be resolved definitionally. I also argued against an overly strict definition of medical need on the grounds that this presupposes, wrongly, that medical intervention should always be a last resort.

## Notes

a. Horne does suggest that this constraint should not be understood as ‘absolute’, mentioning contraception and abortion as possible exceptions.
